# Establishment of the first International Standard for human anti-typhoid capsular Vi polysaccharide IgG

**DOI:** 10.1016/j.biologicals.2018.09.001

**Published:** 2018-11

**Authors:** Sjoerd Rijpkema, Jason Hockley, Alastair Logan, Peter Rigsby, Eleanor Atkinson, Celina Jin, David Goldblatt, Haoyu Liang, Novilia S. Bachtiar, Jae Seung Yang, Akshay Goel, Venkatesan Ramasamy, Marcela F. Pasetti, Andrew J. Pollard, the anti-Vi IgG working group

**Affiliations:** a Division of Bacteriology, National Institute for Biological Standards and Control, Potters Bar, Hertfordshire, EN6 3QG, United Kingdom; b Biostatistics Group, National Institute for Biological Standards and Control, Potters Bar, Hertfordshire, EN6 3QG, United Kingdom; c Oxford Vaccine Group, Department of Paediatrics, University of Oxford and the NIHR Oxford Biomedical Research Centre, Oxford, United Kingdom; d University College London, Great Ormond Street Institute of Child Health, London, United Kingdom; e Institute for Biological Product Control, National Institute for Food and Drug Control, No.2 Tiantan Xili, Beijing, People's Republic of China; f Clinical Trial Department, Surveillance & Clinical Trial Division, Bio Farma, Jl.Pasteur No.28, Bandung, Indonesia; g Clinical Immunology, International Vaccine Institute, SNU Research Park, 1 Kwanak-Ro, Kwanak-Gu, Seoul, Republic of Korea; h R&D, Biological E. Ltd, MN Park, Genome Valley, Shameerpet, Hyderabad, 500078, Telangana, India; i Quality Operations, Bharat Biotech International Ltd, Genome Valley, Shameerpet, Hyderabad, 500078, Telangana, India; j Center for Vaccine Development, University of Maryland Baltimore, 685 West Baltimore Street, Room 480, Baltimore, MD, USA

**Keywords:** International standard, IgG, Typhoid, Vi, Polysaccharide, ELISA, AD, Assay Diluent, BB, Blocking Buffer, CV, coefficient of variation, ECBS, Expert Committee on Biological Standardization, GCV, geometric coefficients of variation, GM, geometric mean, IS, International Standard

## Abstract

Vi capsular polysaccharide (Vi) conjugate vaccines, which can prevent typhoid in infants and young children, are being developed. Comparative immunogenicity studies are facilitated by an International Standard (IS) for human anti-Vi IgG. 16/138, a pool of sera from volunteers which received either Vi conjugate vaccine or plain Vi vaccine, was assessed as an IS alongside U.S. reference reagent Vi-IgG_R1, 2011_. Samples were tested in a commercial ELISA (*n* = 7), a standardised ELISA based on biotinylated Vi (*n* = 7) and in-house ELISAs (*n* = 7). Valid estimates were obtained for the potency of all samples in the commercial ELISA, and the commutability of 16/138 and Vi-IgG_R1, 2011_ was evident for the commercial ELISA and in-house ELISAs based on a coating of Vi and protein. The WHO Expert Committee on Biological Standardization established 16/138 as the first IS for anti-Vi IgG with 100 IU per ampoule and assigned 163 IU per vial of Vi-IgG_R1, 2011_.

## 1 Introduction

Typhoid fever is caused by an infection with *Salmonella enterica* subspecies *enterica* serovar Typhi. *S.* Typhi expresses a capsular polysaccharide, Vi, which is a virulence factor and considered the main protective antigen [1,2]. In developing countries, typhoid disease affects in particular children and young adults and is a considerable cause of morbidity and mortality. A recent study estimated a global incidence of 12 million cases of typhoid and 129,000 deaths each year [3].

Vaccination is the most cost effective preventative strategy to control typhoid fever in endemic areas and in areas where anti-microbial resistant strains reside. However, oral live attenuated Ty21a vaccine and the injectable plain Vi vaccine, which is prequalified by the WHO, are not suitable for use in children under five and two years of age respectively, and plain Vi vaccines are unable to induce a booster response in adults and children [[4], [5], [6]]. Early studies of *Haemophilus influenzae* polysaccharide vaccines showed that the latter issue was successfully addressed by conjugation of the polysaccharide to a carrier protein, usually a bacterial toxoid [7,8]. A prototype typhoid conjugate vaccine (TCV) consisting of Vi conjugated to recombinant *Pseudomonas aeruginosa* exoprotein A (Vi-rEPA) was proven to be immunogenic and induced a booster response in young children [9]. A double-blind randomized clinical trial of Vi-rEPA in 2–5-year olds revealed the vaccine to be safe and efficacious [10]. Vi-rEPA also induced a long lasting anti-Vi IgG response in pre-school age children and infants [11]. Recently, two Vi tetanus toxoid conjugate vaccines (Vi-TT) were licensed in India [12,13]. Currently, 10 TCVs using a variety of carrier proteins for conjugation of Vi are in development: three vaccines are in clinical trials and three vaccines are approaching licensure (personal communication, S. Sahastrabuddhe).

The first international reference serum TYS, a freeze dried anti-Vi serum from a horse immunised with rough and smooth *S* Typhi, was made available in 1935 [1,14]. In 2009, the WHO Expert Committee on Biological Standardization of WHO (ECBS) asked the National Institute for Biological Standards and Control (NIBSC) to produce a human anti-Vi IgG serum standard to replace TYS. In 2010, candidate replacement 10/126, a freeze dried pool of sera from clinical trial volunteers who had been immunised with two doses of a Vi-TT (Typbar TCV™, Bharat Biotech International Ltd, India) was produced [15].

In 2012, a WHO working group discussed the need for a regulatory framework to evaluate TCVs, compare clinical trial studies and analyse the safety, consistency and potency of TCVs by calibrated physicochemical assays and immunoassays. This resulted in the WHO guidelines on the quality, safety and efficacy of TCVs, which confirmed the need for reference materials for Vi and human anti-Vi serum [16,17]. In 2013, Vi PS lot 05 and Vi-IgG_R1, 2011_ were established as U.S. reference reagents for Vi and human anti-Vi IgG respectively. Vi-IgG_R1, 2011_ is a purified fraction of IgG from plasma donations by volunteers vaccinated with Vi-rEPA and contains 33 μg anti-Vi IgG mL^−1^ or 26.6 ELISA Units mL^−1^ [18].

The WHO working group proposed a collaborative study (CS) to evaluate the performance of both Vi-IgG_R1, 2011_ and candidate International Standard (IS) 10/126 to avoid potential confusion in the future [16]. Their performance was consistent in the commercial VaccZyme Human Anti-*Salmonella* typhi Vi IgG ELISA (Binding Site, UK), but results from in-house ELISAs were highly variable [15]. A review by ECBS concluded that reliance on a commercial ELISA combined with the paucity of data about this assay provided insufficient evidence to establish 10/126 as a WHO IS. ECBS requested: a clarification of the inter- and intra-laboratory variation among in-house ELISAs; a new set of anti-Vi positive sera from a clinical trial to establish commutability of both reference reagents; and the establishment of a reliable non-commercial Vi ELISA [15,19].

Previous studies reported poor binding of bacterial polysaccharides such as Vi to the micro-plate surface as a cause for inconsistent ELISA results. This was mitigated by a pre- or co-coating of the polysaccharide with a protein or by chemical modification of the polysaccharide [[20], [21], [22]]. Ferry et al. showed that an ELISA based on biotinylated Vi provided a specific and sensitive screen for anti-Vi IgG in naïve and vaccinated individuals [23]. This observation informed the development of an in-house biotinylated Vi ELISA described here and designated NIBSC ELISA.

In collaboration with the Oxford Vaccine Group (OVG), sera from volunteers immunised with either a WHO prequalified and licensed plain Vi vaccine or a WHO prequalified and licensed Vi-TT conjugate vaccine as described by Jin et al. were obtained [24]. These sera were pooled and freeze dried to produce 16/138 and a quality control (QC) panel made up of individual pre- and post-vaccination sera.

The intended use of 16/138 is to benchmark Vi ELISAs used to evaluate clinical trials to allow a comparison of the immunogenicity of various TCVs. The main aim of the CS was to assess the suitability of 16/138 to serve as a WHO IS for anti-Vi PS IgG (human) in various ELISAs. The second aim was to assign a unitage to 16/138 and a relative unitage to Vi-IgG_R1, 2011_ and 10/126, which remains available at NIBSC. Thirdly, the commutability of 16/138 was assessed with individual anti-Vi PS IgG sera from the QC panel and finally the performance and reproducibility of the NIBSC ELISA alongside the commercial VaccZyme ELISA was assessed across multiple laboratories.

## 2 Materials and methods

### 2.1 Participating laboratories and assay codification

Seven participants, including vaccine manufacturers, national control laboratories and research laboratories from six countries tested the study samples and supplied data for this study (see acknowledgments section for details). Throughout the study, laboratories were identified by a randomly assigned code number to maintain confidentiality. Data were collected and analysed at NIBSC. Six unique plate layouts for the assay runs were provided in the study protocol.

### 2.2 Samples used in the collaborative study

Each participant received a sample set comprising one ampoule of 16/138, coded ampoules (A-F), and one ampoule of Vi-IgG_R1, 2011_, supplied by the Center for Biologics Evaluation and Research (CBER) of the Food & Drug Administration of the Department of Health and Human Services, USA (see Table 1). In addition, essential reagents for the NIBSC ELISA and 6 VaccZyme human anti-*Salmonella* Typhi Vi IgG ELISA kits (Binding Site, UK) were included. These reagents were accompanied by instructions for use, material safety data sheets, and a standard operating procedure for the NIBSC ELISA and manufacturer's instructions for the commercial ELISA. A brief characterisation of the study samples, study codes, NIBSC codes and their reactivity in the VaccZyme ELISA is given in Table 1. All samples tested negative for antibodies to HIV 1/2 and hepatitis C and hepatitis B surface antigen. Freeze dried samples 16/138 and A to F were reconstituted as described in the Instructions for Use. The production and characterisation of Vi-IgG_R1, 2011_ is provided in detail by Szu et al. [18]. This reference reagent was reconstituted as described in the product circular. Reagents for the NIBSC ELISA consisted of one ampoule of 5 mg purified streptavidin from *Streptomyces avidinii* (S4762, Sigma-Aldrich), one ampoule of 1 mg freeze dried biotinylated Vi derived from *Citrobacter freundii* (NIBSC12/244; Innova Biosciences), and one ampoule of goat anti-Human IgG horse radish peroxidase (HRP) conjugate (A8667, Sigma-Aldrich). All samples were sent on dry ice, with the exception the VaccZyme ELISA kits sent at 2–8 °C.

**Table 1:** Characterisation of samples used in this study.

Study code	NIBSC code	Description	Reactivity in the VaccZyme ELISA
16/138	–	Pooled Anti-Vi IgG sera from 16 volunteers	631 U mL^−1^^a^
Vi-IgG_R1, 2011_	–	Purified Anti-Vi IgG from 5 volunteers	1280 U mL^−1^^b^
A	10/126	Pooled Anti-Vi IgG sera from 9 volunteers	842 U mL^−1^^c^
B	16/150	Post-Typbar TCV vaccination serum	350 U mL^−1^^c^
C	16/138	Pooled Anti-Vi IgG sera from 16 volunteers	631 U mL^−1^^a^
D	16/144	Post-Vi PS vaccination serum	345 U mL^−1^^c^
E	16/146	Post-Vi PS vaccination serum	524 U mL^−1^^c^
F	16/140	Pre-Vaccination serum	22 U mL^−1^^c^

a Based on an estimate from 3 runs.

b Based on an estimate from 4 runs.

c Based on an estimate from one run.

### 2.3 Characterisation of 16/138

Sera from vaccinated volunteers, as described below, were supplied by OVG. Ethical approval was obtained from South Central Oxford A Ethics Committee (14/SC/1427) and the protocol was approved by the Medicines and Healthcare Products Regulatory Agency (Eudract 2014-002978-36). The Human Materials Advisory Committee of NIBSC (16/004SR) approved the use of these sera as a source for 16/138.

**Fig. 1:**
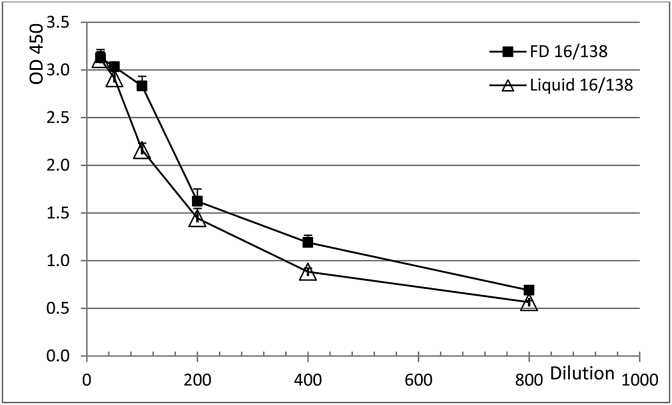
Titration of anti-Vi IgG in liquid (△) and freeze dried (■) formulations of 16/138 by the NIBSC ELISA.

In brief, consenting healthy volunteers (age range 18–57 years) received one dose of either plain Vi (Typhim Vi™, Sanofi Pasteur; Vi-PS; *n* = 15) or Vi-TT (Typbar TCV™; TCV; *n* = 14). Sera were collected prior to vaccination and 4–6 weeks post-vaccination. This vaccination schedule closely resembled the schedule used in an assessment of vaccine efficacy using a controlled human infection model in which both plain Vi-PS and TCV were evaluated [24]. Frozen sera were transferred to NIBSC and stored at −80 °C until further use. Individual pre- and post-immunisation sera were tested for anti-Vi IgG in the VaccZyme ELISA and the NIBSC ELISA. Post-vaccination sera which showed a >3-fold increase in ELISA Units in both assays were selected as source material for 16/138 (results not shown). Seven sera from volunteers vaccinated with Vi-PS and 9 sera from volunteers vaccinated with TCV were pooled. The pool of approximately 3 L was dispensed in 1.0 mL aliquots into glass ampoules coded 16/138. The mean fill weight for 98 ampoules was 1.007 g (CV of 0.21%). On the same day, freeze-drying under vacuum started and was completed after four days. Ampoules were back filled with pure N_2_ (moisture content <10 ppm). Residual moisture, measured by the Karl-Fischer method, for 12 ampoules was 0.5246% (CV of 6.92%). An integrity test was not performed; however, the oxygen level in the headspace of 12 ampoules was checked and found to be 0.39% (CV of 29.97%). This implies these ampoules passed a test for integrity because the presence of cracks would be associated with an oxygen level of around 20%, similar to that found in the atmosphere. Twelve ampoules were rejected during the production process, 30 ampoules were held for accelerated degradation studies and 2551 ampoules were stored at −20 °C. The fill of 16/138 met the WHO criteria for ISs [25]. The ampoules are available for distribution by NIBSC. The effect of freeze drying on levels of specific anti-Vi IgG was determined by NIBSC ELISA in liquid and freeze dried samples of 16/138. The freeze-drying process did not affect the anti-Vi IgG titre (see Fig. 1).

### 2.4 Characterisation of coded sera

Twelve individual pre- and post-vaccination sera of volunteers, immunised with Vi-PS or TCV and representing a range of anti-Vi IgG levels, were used to assemble a serum panel. Sera were dispensed in 80 ampoules as 1.0 mL aliquots, freeze dried and stored at −20 °C until further use. Four sera were selected as test samples coded B, D, E and F (see Table 1).

### 2.5 Characterisation of 10/126

Approximately 1 L of human serum was collected from 9 consenting volunteers (24–30 years old) who had received two doses of TCV as part of a phase II clinical trial (see Table 1 [15]). The vaccinations were given 4 weeks apart and sera were collected at week 6 after the booster and frozen. The use of source material for candidate IS 10/126 was approved by the Human Materials Advisory Committee of NIBSC 10_002SR. At NIBSC, samples were thawed and pooled. The serum pool was dispensed as 0.5 mL aliquots into glass ampoules coded 10/126. The mean fill weight for 78 ampoules was 0.5181 g (CV of 0.19%). On the same day, freeze-drying under vacuum started and was completed after four days. Ampoules were back filled with pure N_2_ (moisture content <10 ppm). Residual moisture, measured by the Karl-Fischer method, for 6 ampoules was 0.23% (CV of 17.60%). An integrity test was not performed; however the oxygen level in the headspace of 12 ampoules was checked and found to be 0.59% (CV of 29.55%). Twenty-six ampoules were rejected during the production process, 44 ampoules were held for accelerated degradation studies and 1654 ampoules are stored at −20 °C and available for distribution by NIBSC. Freeze drying had no effect on the titre of IgG directed against Vi in 10/126 [15].

### 2.6 ELISAs

Study samples were tested in the VaccZyme ELISA and eight in-house ELISAs. The ELISA code and a brief description of the coating procedure are given in Table 2. Two ELISA formats were made available to all participants: the VaccZyme ELISA based on native *S Typhi* Vi, and the NIBSC ELISA based on a streptavidin-biotinylated *C freundii* Vi complex. All participants performed the NIBSC ELISA, the commercial Vacczyme ELISA as well as their in-house ELISA. ELISA procedures are given below. Six different plate layouts were provided to the participants and samples were tested repeatedly in 6 runs, except for Laboratory 2 (4 runs for the Vacczyme ELISA and 2 runs for their in-house ELISA) and Laboratory 6 (5 runs for the Vacczyme ELISA). Each participant was asked to prepare starting dilutions of sera and reference reagents of 1:100 in appropriate assay diluents, with the exception of 16/138, which had a starting dilution of 1:25. A description of the ELISA procedure accompanied the raw data returned by the participant for analysis.

**Table 2:** Format of Vi ELISAs used by participants.

Laboratory code	ELISA method		Vi characteristics		Antigen coating procedure
	Name	Format	Status	Biological origin	
1, 2, 3, 4, 5, 6,7	VaccZyme	Indirect	–	*S* Typhi	Non-disclosed procedure
1, 2, 3, 4, 5, 6,7	NIBSC	Capture	Biotinylated	*C freundii*	Streptavidin coat to bind biotinylated Vi
1	In-house	Indirect	Naive	*S* Typhi	Pre-coat with poly–l-Lysine to bind Vi
2	In-house	Indirect	Naive	*C freundii*	Vi coat only
3	In-house	Indirect	Naive	*C freundii*	Vi coat only
4	In-house	Indirect	Naive	*S* Typhi	Pre-coat with poly–l-Lysine to bind Vi
5	In-house	Indirect	Naive	*C freundii*	Mix coat with mHSA and Vi
6	In-house	Capture	Biotinylated	*S* Typhi	Streptavidin coat to bind biotinylated Vi
7	In-house	Indirect	Naive	*S* Typhi	Vi coat only

#### 2.6.1 VaccZyme ELISA

The VaccZyme ELISA was carried out according to the manufacturer's instructions (Binding Site). The VaccZyme ELISA is based on Vi of *S.* Typhi. Five calibrators, covering a range from 7.4, 22.2, 66.7, 200 and 600 EU mL^−1^, and a high and a low positive control were included in each run. The assay has a sensitivity of 7.4 EU mL^−1^. Plates were read at OD_450nm_.

#### 2.6.2 NIBSC ELISA

This in-house ELISA, developed at NIBSC, follows the procedure described by Ferry et al. with minor modifications [23]. The plate surface is coated with streptavidin to capture biotinylated Vi from *C freundii* (NIBSC 12/244; Innova Biosciences). For this purpose, approximately 20 ampoules with a solution of 1 mg biotinylated Vi from *C freundii* and 10 mg Trehalose (a cryopreservant) were freeze dried and stored at −20 °C at NIBSC until distribution among participants. Wells of a micro-titre plate (Nunc MaxiSorp) were pre-coated with 100 μL of 5 μg Streptavidin mL^−1^ (Sigma Aldrich, S4762) in sterile water and incubated uncovered overnight at 37 °C. The content of one ampoule with freeze dried biotinylated Vi was reconstituted overnight at 4 °C with 1 mL Mill-Q water. On the following day, 3 μg biotinylated Vi mL^−1^ was prepared from stock in PBS, 100 μL was added to wells of a streptavidin-coated plate and incubated for 3 h at 37 °C. Plates were washed three times and 250 μL Blocking Buffer 1 (BB1: 10% non-fat dry milk (w/v) in PBS (pH 7.3–7.5) was added to each well. Plates were incubated for 2 h at 4 °C and washed 3x with PBS-Tween 0.05% (PBS-T). Sera and reference reagents were diluted in Assay Diluent 1 (AD1; 0.05% Tween-20 in BB1). 200 μL each of starting dilutions was added to appropriate wells and 100 μL AD1 was added to the remaining wells. Doubling dilutions were performed on the plate. Plates were covered and incubated for 2 h at RT (range 20–25 °C) and washed 3x with PBS-T. 100 μL of HRP conjugated goat anti-human IgG (Sigma Aldrich, A8667) diluted 1:10,000 in AD1 was added to each well. Plates were covered, incubated for 1 h at RT and washed 3x with PBS-T. 100 μL of TMBlue substrate (Leinco Technologies) was added to each well. Plates were developed in the dark for 10 mins and the reaction was stopped with 50 μL H_2_SO_4_ per well. Plates were read at OD_450nm_.

#### 2.6.3 In-house ELISA 1

The ELISA carried out by Laboratory 1, is a modification of the ELISA described by Szu et al. [18]. Nunc Maxisorp plates are pre-coated of with Poly-l-Lysine hydrobromide (Sigma, P1399) followed by a coat with Vi from *S.* Typhi (SK chemicals, CT1S0153) [26]. 100 μL of 10 μg poly-l-Lysine mL^−1^ in PBS was added to the wells, incubated at RT for 2 h and washed 6x with 0.85% NaCl and 0.1% Brij35 in ultrapure water (NaCl-Brij 35). 100 μL of 2 μg Vi mL^−1^ in PBS was added to the wells. Plates were incubated overnight at 37 °C and washed 6x with NaCl-Brij 35. 250 μL BB2 [1% BSA in PBS) was added to each well. Plates were incubated for 1 h at 37 °C and washed six times. Dilutions of test samples were prepared in AD2 (BB2 with 0.1% Brij35). Then 100 μL of diluted tested sample was dispensed to appropriate wells of the plate. Samples were incubated at 37 °C for 1 h and washed 6x with NaCl-Brij 35. Then 100 μL of alkaline-phosphatase (AP) conjugated mouse anti-human IgG Fc (Abcam, AB99764) diluted 1:2000 in AD2 was added to each well, incubated at 37 °C for 1 h and washed 6x with NaCl-Brij 35. 100 μL 4-Nitrophenyl Phosphate disodium salt hexahydrate substrate (Sigma) in Tris/3 mM Mg-buffer (pH 9.8) was added per well and incubated at RT in dark for 1 h, followed by the addition of 50 μL 3 M NaOH stop solution to each well. The plate was read at 405 nm and 490 nm. Background OD_490_ was subtracted from OD_405_.

#### 2.6.4 In-house ELISA 2

The procedure carried out by Laboratory 2 uses Vi from *C. freundii* produced in–house. Wells were coated with 100 μL of 4 μg Vi mL^−1^ diluted in PBS and incubated overnight at 2–8 °C. Nunc MaxiSorp plates were washed 1x with PBS-T using a plate washer and plates were tapped dry on tissue paper. Wells were blocked with 200 μL BB3 (PBS + 5% skimmed milk) and incubated at RT for 1 h. Plates were washed 3x with PBS-T. Sera were diluted in AD3 (PBS-T + 0.1% BSA) and 100 μL of each dilution was added to wells in duplicate. Plates were incubated at RT for 2 h and washed 3x with PBS-T. Goat anti Human IgG-AP (Sigma-Aldrich, A9544) was diluted 1:15,000 in AD3 and 100 μL was added to each well. Plates were incubated at RT for 1 h. Plates were washed 5x with PBS-T. 4-Nitrophenyl Phospate liquid substrate (Sigma-Aldrich, N-7653) was warmed to RT and 100 μL was added per well. Plates were incubated at RT for 1 h. The reaction was stopped by adding 100 μL of 2 M NaOH to each well. The plate was read at 405 nm and 490 nm. Background OD_490_ was subtracted from OD_405_.

#### 2.6.5 In-house ELISA 3

The procedure carried out by Laboratory 3 is based on the ELISA described by Szu et al. and uses Vi lot 05 supplied by CBER [18]. Wells (Nunc Maxisorp) were coated with 100 μL of 2 μg Vi mL^−1^ diluted in PBS. Plates were covered, incubated overnight at 25 °C, washed 2x with NaCl-Brij and shaken dry. Wells were blocked with BB2 for 2 h at 25 °C, washed 2x with NaCl-Brij and shaken dry. Starting dilutions of test samples were prepared in AD2. Wells of the first row received 200 μL of the starting dilution and all other wells received 100 μL AD2. Top down dilutions were prepared by transfer of 100 μL of the diluted sample to subsequent wells. Plates were covered, incubated overnight at 25 °C, washed 2x with NaCl-Brij and shaken dry. Hundred μL of AP conjugated goat anti-human IgG (Sigma) diluted 1:5000 in AD2 was added to each well and incubated at 37 °C for 4 h. Plates were washed 2x with NaCl-Brij, shaken dry and 100 μL of 4-Nitrophenyl Phosphate disodium salt hexahydrate substrate (Sigma) in Tris/3 mM Mg-buffer (pH 9.8) was added to each well and incubated at RT for 10–30 mins. Plates were read at OD_405nm_.

#### 2.6.6 In-house ELISA 4

The procedure carried out by Laboratory 4 is identical to the procedure for in-house ELISA 1; except for the use of an AP conjugated mouse anti-human IgG Fc (Abcam, AB99764) diluted 1:5000.

#### 2.6.7 In-house ELISA 5

The procedure carried out by Laboratory 5 is based on Arakere and Frasch with some modifications [23]. The plate surface is coated with methylated Human Serum Albumin (mHSA, NIBSC 12/176) followed by a coat of Vi from *C freundii* (NIBSC 12/244). In brief, 100 μL of a mixture consisting 5 μg Vi mL^−1^ and 5 μg mHSA mL^−1^ in PBS was used to coat wells of a microtiter plate (Nunc MaxiSorp). The plate was incubated overnight at 4 °C and washed 3x with PBS-T. Wells were blocked with 250 μL BB1 for 2 h at 4 °C and washed 3x with PBS-T. Sera and reference reagents were diluted in AD1. 200 μL each of starting dilutions was added to appropriate wells and 100 μL AD1 was added to the remaining wells. Doubling dilutions were performed on the plate. Plates were covered and incubated for 2 h at RT and washed three times. 100 μL HRP conjugated goat anti-human IgG (Sigma) diluted 1:10,000 in AD1 was added to each well. Plates were covered, incubated for 1 h at RT and washed 3x with PBS-T. 100 μL of TMBlue substrate (Leinco Technologies) was added to each well. Plates were developed in the dark for 10 mins and the reaction was stopped with 50 μL 2 M H_2_SO_4_ per well. Plates were read at OD_450nm_.

#### 2.6.8 In-house ELISA 6

The procedure carried out by Laboratory 6 is a minor modification of the ELISA described by Ferry et al. [23]. The plate surface is coated with streptavidin to capture biotinylated Vi from *S* Typhi (Fina Biosolutions LLC). In brief, streptavidin from *S avidinii* (Sigma, S4762) was prepared in a solution of 3 μg mL^−1^ and 100 μL was added to each well of flat bottom microtiter plates (Nunc Maxisorp) and incubated uncovered at 37 °C overnight, allowing the solution to evaporate to dryness. Pre-coated plates were stored at 4 °C until used for coating. For antigen coating, 2 μg mL^−1^ of biotinylated *S*. Typhi Vi PS produced locally was prepared in PBS (pH 7.4) and 100 μL was added to the wells of pre-treated plates. Plates were incubated for 3 h at 37 °C and washed with PBS-T and wells were blocked overnight with 250 μL PBS containing 10% non-fat dry milk at 4 °C. Plates were washed 6x with PBS-T with two mins soaking period in between washes. Sera and reference reagents were diluted in AD1 and added to the plates. All plates were incubated for 1 h at 37 °C. Following incubation and washing as described above, plates were incubated with HRP-labelled goat anti-Human IgG (Jackson Immunoresearch, 109-035-008) diluted in AD1 for 1 h at 37 °C. The plates were washed 6x with PBS-T, and 100 μL of TMB Microwell Peroxidase Substrate (KPL, SeraCare Life Sciences Inc) was added to each well, incubating for 15 mins in the dark (with agitation). The reaction was stopped by adding 100 μL of 1 M Phosphoric acid per well. Plates were read immediately at OD_450nm_.

#### 2.6.9 In-house ELISA 7

The procedure carried out by Laboratory 7 is a modification of the ELISA by Szu et al. [18]. The plate surface is coated with directly with Vi PS from *S*. Typhi (lot 201308, Wuhan Institute of Biological Products Co. Ltd). Wells (Costar, 2592) were coated with 100 μL of 2 μg Vi mL^−1^ in PBS diluted from 1 mg mL^−1^ in Milli-Q. Plates were covered, incubated overnight at 4 °C, washed 5x with 1% Brij L23 in 0.8% NaCl (NaCl-Brij 23). Wells were blocked with BB2 for 2 h at 37 °C, washed 5x with NaCl-Brij 23 and shaken dry. Starting dilutions of test samples were prepared in BB2 with 0.1% Brij L23 (AD4). Wells of the first row received 200 μL of the starting dilution and all other wells received 100 μL AD4. Top down dilutions were prepared by transfer of 100 μL of the diluted sample to subsequent wells. Plates were covered, incubated overnight at 25 °C, washed 5x with NaCl-Brij 23 and shaken dry. Hundred μL of AP conjugated goat anti-human IgG (Southern Biotech, 2040-04) diluted 1:2000 in AD4 was added to each well and incubated at 37 °C for 2 h. Plates were washed 5x with NaCl-Brij 23 and shaken dry and 100 μL of 4-Nitrophenyl Phosphate disodium salt hexahydrate substrate (Sigma, 71768) in Tris/3 mM Mg-buffer (pH 9.8) was added to each well and incubated at RT for 90–120 mins, then stopped by 50 μL 3 M NaOH. Plates were read at OD_405nm_.

### 2.7 Statistical analysis

Raw data from all participants were analysed at NIBSC using a four-parameter logistic model (sigmoid curves) or a parallel line model. Assay responses were log transformed and analysis was performed using EDQM CombiStats Software Version 5.0 [27]. Fitted models (Sigmoid Curve or Parallel Line) were used to estimate the potency of coded samples A-E relative to candidate IS 16/138 and U.S. reference reagent Vi-IgG_R1, 2011_ and the potency of the two reference standards relative to each other. Parallelism of dose-response relationships was concluded if the slope ratio calculated by CombiStats was within the range 0.80–1.25 and no relative potency was reported where this was not the case.

All mean results shown in this report are unweighted geometric means (GM). Variability has been expressed using geometric coefficients of variation (GCV = {10^s^-1} × 100% where s is the standard deviation of the log_10_ transformed estimates). Individual assay estimates of relative potency were log transformed and a mixed effects model used to determine intra-laboratory and inter-laboratory variance components (also expressed as %GCV) for each sample in NIBSC and VaccZyme ELISAs.

Further assessment of agreement in geometric mean results for each pair of laboratories was performed by calculating Lin's concordance correlation coefficient with log transformed data, although should be noted that these values are only based on a small number of samples (*n* = 6) in each case. Calculations were performed using the R package ‘DescTools’.

## 3 Results

### 3.1 ELISA performance

All participants reported positive results for 16/138, Vi-IgG_R1, 2011_ and samples A-E in all ELISAs, whereas sample F gave negative results in most ELISAs (See Table 3, Table 4). In the majority of cases, valid estimates were obtained for the potency of coded samples A-E relative to 16/138 or U.S. reference reagent Vi-IgG_R1, 2011_ and for the relative potency of the two reference reagents to each other.

**Table 3:** Potency estimates relative to 16/138 for U.S. reference reagent Vi-IgG_R1, 2011_ and coded samples A to E.

Method	Sample	GM^a^								Overall GM	Inter-lab GCV^b^	Intra-lab GCV
		Laboratory code	1	2	3	4	5	6	7			
NIBSC ELISA	Vi-IgG_R1, 2011_		2.80	–	–	3.50	2.61	3.75	1.80	2.80	**31%**	22%
	A		1.34	–	–	1.47	1.62	1.70	1.15	1.44	16%	10%
	B		0.73	–	–	0.77	0.74	1.03	0.73	0.79	15%	11%
	C		0.85	–	–	0.92	0.90	1.21	1.07	0.98	15%	13%
	D		0.46	–	–	0.50	0.66	0.25	0.25	0.39	**54%**	20%
	E		0.33	–	–	0.39	0.38	0.43	0.60	0.42	**25%**	10%
VaccZyme ELISA	Vi-IgG_R1, 2011_		1.60	2.02	1.60	1.59	1.39	1.63	–	1.63	11%	13%
	A		0.84	1.04	1.18	1.09	1.23	1.21	–	1.09	15%	10%
	B		0.27	0.48	0.47	0.41	0.31	0.39	–	0.38	24%	9%
	C		0.70	0.97	1.01	1.08	0.94	1.22	–	0.97	20%	11%
	D		0.21	0.18	0.25	0.31	0.31	0.29	–	0.25	24%	14%
	E		0.44	0.60	0.65	0.71	0.66	0.69	–	0.62	19%	13%
In-house ELISA	Vi-IgG_R1, 2011_		1.88	–	–	1.89	1.70	5.18	–	–	–	–
	A		0.93	–	–	1.17	1.09	1.51	–	–	–	–
	B		0.46	–	–	0.41	0.53	1.25	–	–	–	–
	C		1.04	–	–	1.01	0.90	1.15	–	–	–	–
	D		0.22	–	–	0.33	0.23	0.14	–	–	–	–
	E		0.68	–	–	0.66	0.47	0.42	–	–	–	–

-: No data entered.

GCV≥25%: Font in Bold.

a GM: Geometric Mean.

b GCV: Geometric Coefficient of Variation.

**Table 4:** Potency estimates for sample F relative to 16/138.

Method	GM^a^						
Laboratory code	1	2	3	4	5	6	7
NIBSC ELISA	0.05	0.10	*0.89*^b^	0.06	0.04	0.10	0.07
VaccZyme ELISA	0.02	0.02	0.02	0.02	0.02	0.02	0.01
In-house ELISA	0.03	0.36	*1.14*^b^	0.02	0.01	0.02	0.08

a Geometric Mean.

b Italicized entries represent original data from the participant not calculated by NIBSC.

For inclusion in further analysis, raw ELISA data had to meet criteria for linearity, parallelism and an intra-laboratory GCV of <25%. Results of six ELISAs were not included for the following reasons: the NIBSC ELISA of Laboratory 2 showed poor repeatability in relative potency estimates with an average GCV >75% and their in-house ELISA had insufficient valid data to calculate relative potencies; the results of the NIBSC ELISA of Laboratory 3 and their in-house did not allow potency estimates to be calculated by parallel line analysis, because the dose-response relationships showed high OD values with no plateau in dose-response and no linear section covering more than two dilutions; the in-house ELISA of Laboratory 7 had a high level of assay invalidity (68%) and the VaccZyme ELISA showed poor repeatability in relative potency estimates (average GCV >75%). Results of these assays are shown in supplementary Tables A1 and A2 which contain all individual potency estimates [28].

**Fig. 2:**
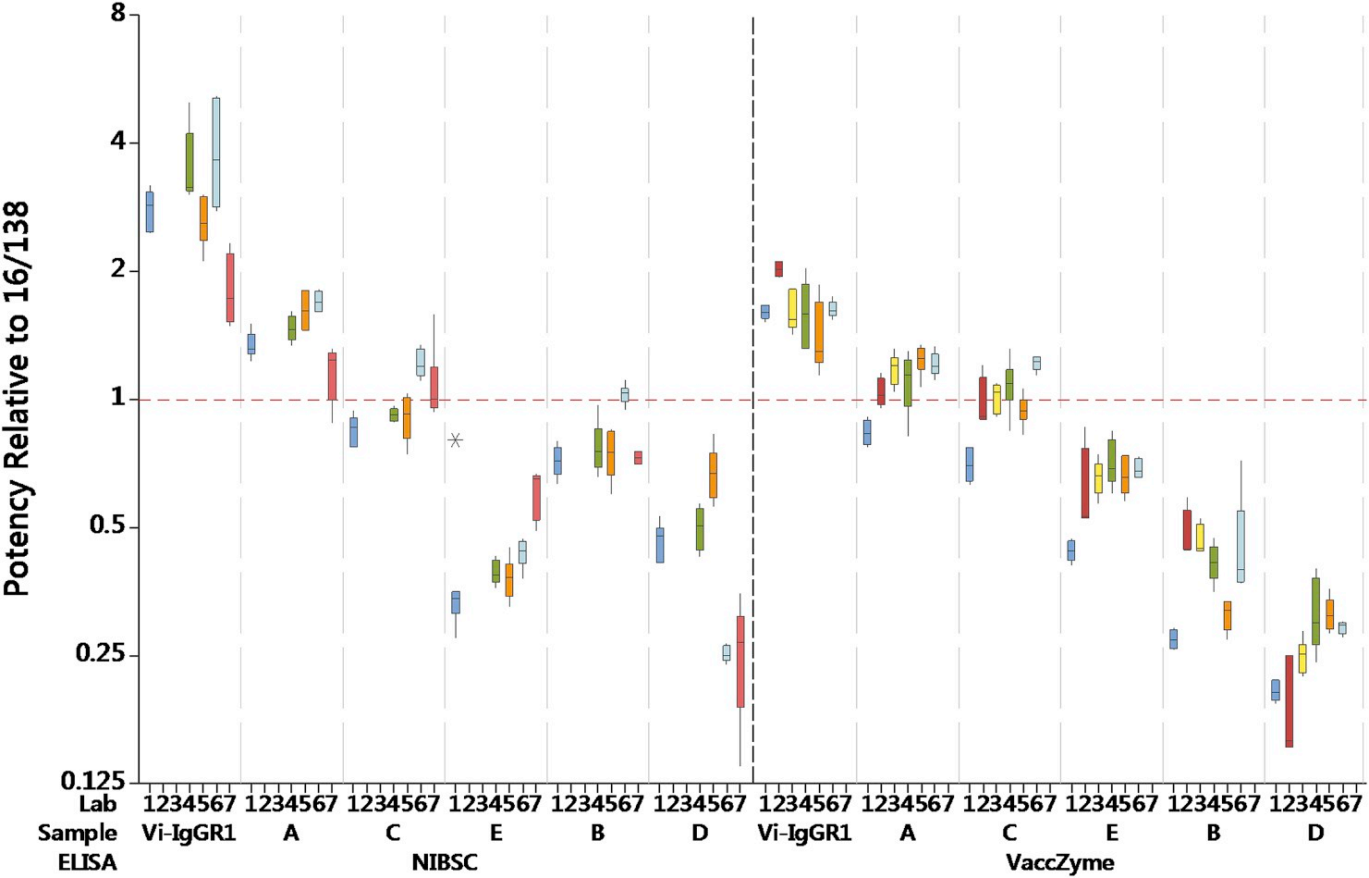
Boxplot of potencies of samples Vi-IgG_R1, 2011_ and A to E relative to 16/138 in NIBSC and VaccZyme ELISAs.

The relative potency of 16/138 and sample C (a coded duplicate of 16/138) was generally close to the expected value of 1.00 in most ELISAs (see Table 3, Fig. 2 and supplementary Table A1), although a systematic bias was observed in some cases (e.g. VaccZyme ELISAs of Laboratories 1 and 6). The reasons for this are unclear as the laboratories followed the study protocol in all cases and these were not excluded from the analysis presented in Table 3, Table 5.

**Table 5:**
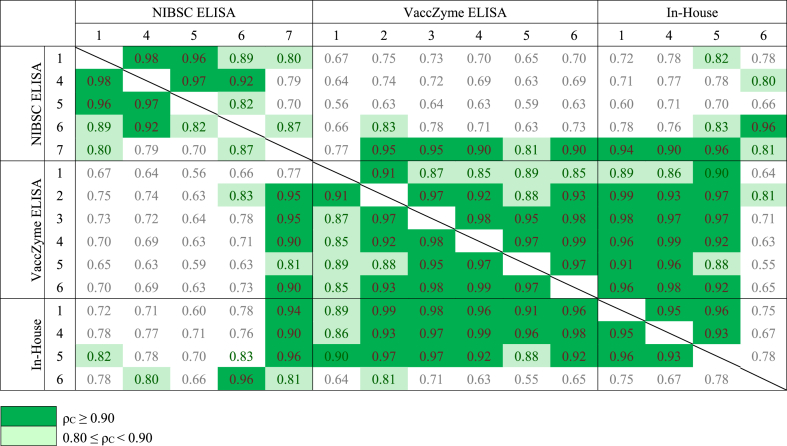
Concordance correlation coefficients (ρ_C_) for log potencies of Vi-IgG_R1, 2011_ and samples A to E relative to 16/138.

Potency estimates for the pre-vaccination serum sample F relative to 16/138 are given in Table 4. Most laboratories found sample F to be negative for anti-Vi IgG (relative potency ≤0.1). A high relative potency value for sample F was recorded by Laboratory 3 for the NIBSC ELISA and their in-house ELISA and by Laboratory 2 for their in-house ELISA.

### 3.2 Comparison of NIBSC and VaccZyme ELISAs

Table 3 summarises the GM potencies per laboratory, overall GM potencies, intra-laboratory GCVs and inter-laboratory GCVs. In Fig. 2, the boxplots show the potency estimates of the study samples in NIBSC and VaccZyme ELISAs relative to 16/138. Inter-laboratory and intra-laboratory variability, given in Table 3, was generally higher in the NIBSC ELISA (GCV∼26% using 16/138, ∼35% using Vi-IgG_R1, 2011_) compared to VaccZyme (∼19% using 16/138, ∼23% using Vi-IgG_R1, 2011_). A similar outcome was observed when Vi-IgG_R1, 2011_ was used as reference [28].

For the study samples, no agreement in relative potencies was observed between the NIBSC and VaccZyme ELISAs (See Table 3). The coded samples are shown in decreasing order of potency in the VaccZyme ELISA in Fig. 2. Note that a different ranking of the coded samples is observed for the NIBSC ELISA.

### 3.3 Concordance between NIBSC, VaccZyme and in-house ELISAs

**Fig. 3:**
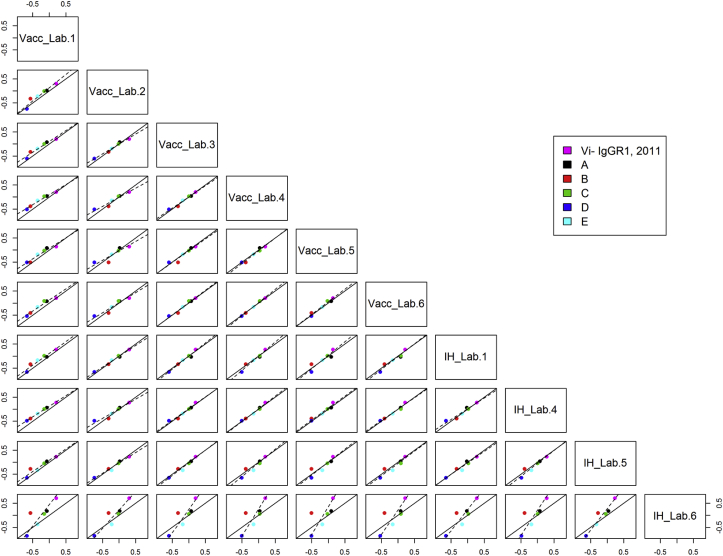
Scatterplots of log potencies relative to 16/138 in VaccZyme (Vacc) and in-house ELISAs (IH) for individual lab pairs; solid line indicates agreement, dashed line indicates fitted Deming regression.

**Fig. 4:**
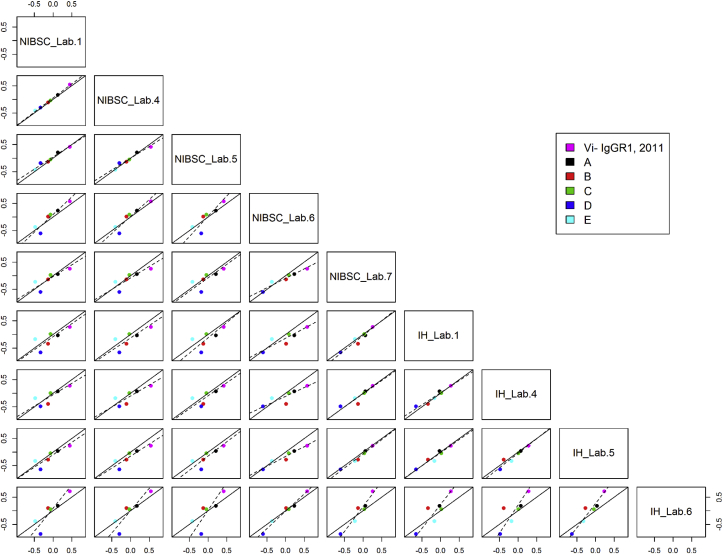
Scatterplots of log potencies relative to 16/138 in NIBSC and in-house ELISAs (IH) for individual lab pairs; solid line indicates agreement, dashed line indicates fitted Deming regression.

The concordance correlation coefficients for all individual assay pairs using 16/138 as reference standard is shown in Table 5. Values of >0.80 have been highlighted to show where better agreement between assays was observed. Scatterplots for assay pairs with fitted Deming regression lines included on each plot are shown in Fig. 3, Fig. 4. Agreement between the VaccZyme ELISA and in-house ELISAs performed by Laboratories 1, 4 and 5 is evident from Fig. 3, Fig. 4 and Table 5. No or limited concordance was seen for the VaccZyme ELISA and the NIBSC ELISA in Table 5 and Fig. 3, Fig. 4. A similar outcome was observed when Vi-IgG_R1, 2011_ was used as reference standard [28]. Individual potency estimates relative to 16/138 and Vi-IgG_R1, 2011_ obtained by in-house ELISAs 1, 4 and 5 revealed that data produced by ELISA 1 and 4 are more robust in terms of parallelism and intra-test GCV compared to data from ELISA 5 (supplementary Tables A1 and A2 [28]).

### 3.4 Potency estimates for Vi-IgG_R1, 2011_ and 10/126 relative to 16/138

**Fig. 5:**
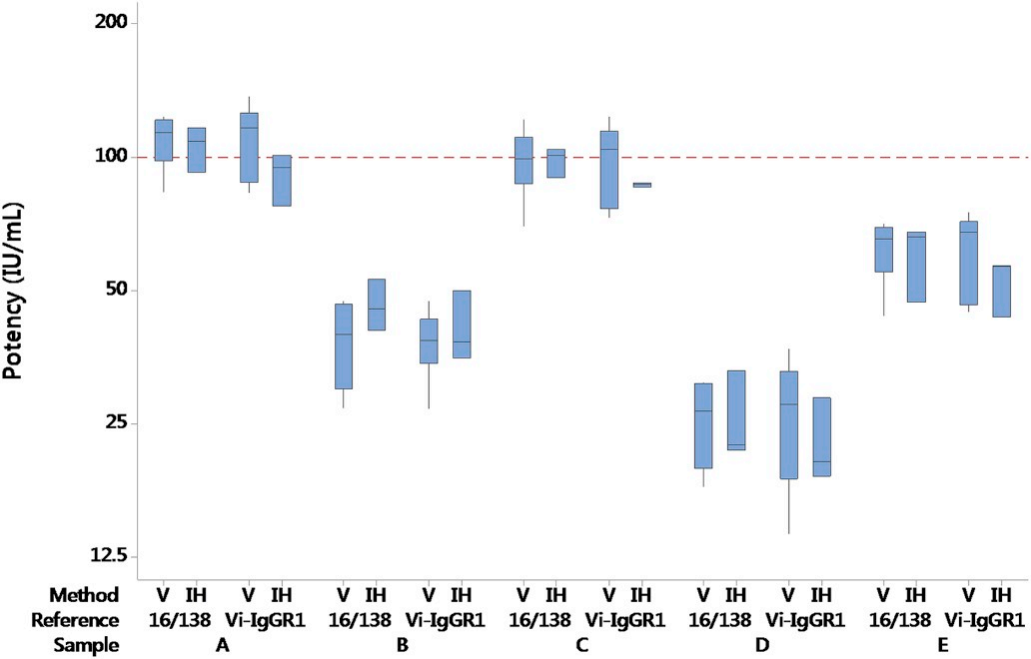
Boxplot of log potencies of study samples calculated relative to 16/138 (assigned 100 IU per ampoule) or Vi-IgG_R1, 2011_ (assigned 163 IU per vial) in VaccZyme ELISAs (V) and in-house ELISAs by Laboratories 1, 4 and 5 (IH).

Potency estimates were calculated for Vi-IgG_R1, 2011_ and 10/126 relative to 16/138 are summarised in Table 6. The overall GM potency of Vi-IgG_R1, 2011_ calculated relative to 16/138 in the VaccZyme ELISA is 1.63 (95% confidence limits 1.44 to 1.85; *n* = 6), which is equivalent to 163 IU mL^−1^ if 16/138 is assigned an arbitrary unitage of 100 IU per ampoule (or 100 IU mL^−1^). Inclusion of the in-house ELISAs by Laboratories 1, 4 and 5 gives an estimate of 1.69 (95% confidence limits 1.55 to 1.85; *n* = 9). As shown in Table 6, the overall values were not affected by the inclusion of sample C (coded duplicate of 16/138) in these calculations. The level of agreement in potency estimates obtained for coded samples A-E when calculated relative to 16/138 (assigned 100 IU per ampoule) or relative to Vi-IgG_R1, 2011_ (assigned 163 IU mL^−1^) is shown in Fig. 5.

**Table 6:** Potency estimates for U.S. reference reagent Vi-IgG_R1, 2011_, the coded duplicate of 16/138 and 10/126 relative to 16/138.

Method	Lab code	Sample C (16/138)	Vi-IgG_R1, 2011_	Sample A (10/126)
VaccZyme ELISA	1	0.70	1.60	0.84
	2	0.97	2.02	1.04
	3	1.01	1.60	1.18
	4	1.08	1.59	1.09
	5	0.94	1.39	1.23
	6	1.22	1.63	1.21
In-house ELISA	1	1.04	1.88	0.93
	4	1.01	1.89	1.17
	5	0.90	1.70	1.09
GM^a^ by VaccZyme ELISA (95% CI^b^)			1.63 (1.44–1.85)	1.09 (0.94–1.27)
GM by all ELISAs (95% CI)			1.69 (1.55–1.85)	1.08 (0.98–1.19)
GM by VaccZyme ELISA based on potency relative to both 16/138 and sample C ^(^95% CI)			1.65 (1.41–1.92)	1.10 (1.01–1.21)
GM by all ELISAs based on potency relative to both 16/138 and sample C ^(^95% CI)			1.71 (1.55–1.89)	1.09 (1.01–1.18)

a Geometric Mean.

b Confidence Interval.

The GM potency of 10/126 calculated relative to 16/138 in the VaccZyme ELISA is 1.09 (95% confidence limits 0.94 to 1.27; *n* = 6) or 54 IU per ampoule equivalent to 109 IU mL^−1^, if 16/138 is assigned a unitage of 100 IU per ampoule. Inclusion of the in-house ELISAs by Laboratories 1, 4 and 5 gives an estimate of 1.08 (95% confidence limits 0.98 to 1.19; *n* = 9). The GM potency of 10/126 calculated relative to Vi-IgG_R1, 2011_ is 0.67 in the VaccZyme ELISA (*n* = 6), which is close to the value of 0.65 reported previously [15].

### 3.5 Stability studies

Freeze dried samples of 16/138 were stored for 8.8 months at −20 °C and at elevated temperatures: +4 °C, +20 °C, +37 °C and +45 °C. Two ampoules of 16/138 exposed to each temperature were tested in duplicate by Laboratory 5 in the VaccZyme ELISA, the NIBSC ELISA and the mHSA Vi ELISA. The potencies relative to samples stored at −20 °C are given in Table 7.

**Table 7:** Thermal stability of 16/138 stored at elevated temperatures relative to storage at −20 °C.

Exposure		Method	Anti-Vi IgG potency relative to −20 °C^a^		
Duration	Temperature		Vial 1	Vial 2	GM
8.8 months	+4 °C	NIBSC ELISA	0.90	0.86	0.88
	+20 °C		0.83	0.81	0.82
	+37 °C		0.74	0.70	0.72
	+45 °C		0.39	0.38	0.38
	+4 °C	VaccZyme ELISA	0.97	1.03	1.00
	+20 °C		0.95	1.05	1.00
	+37 °C		0.82	0.96	0.89
	+45 °C		NL^b^	0.73	0.73

a Data produced by Laboratory 5.

b Non linear.

The results were analysed by DEGTEST-R (in-house NIBSC software) and used to fit Arrhenius equations relating the degradation rate to absolute temperature assuming first-order decay and hence predict the degradation rates when stored at −20 °C [29]. Only results of the VaccZyme ELISA gave a predicted loss of activity for 16/138 stored at −20 °C of 0.002% per year. The data from the NIBSC ELISA were analysed but the chi-squared test for goodness of fit showed these not to fit the model (results not shown). The relative potencies are shown for information only. Data from the mHSA Vi ELISA could not be analysed due to the non-linearity of the dose responses curves (results not shown).

## 4 Discussion

The results of the collaborative study demonstrate that 16/138 is suitable as an IS for anti-Vi IgG serum (human) in various ELISA formats used in Vi serology, where the commutability of 16/138 with coded test samples A-E and Vi-IgG_R1, 2011_ was evident in the VaccZyme ELISA and in-house ELISAs by Laboratories 1, 4 and 5.

The outcomes for assay validity, variability in potency estimates, agreement between methods and laboratories are similar for both 16/138 and Vi-IgG_R1, 2011_. Based on the commercial VaccZyme ELISA, ECBS assigned an arbitrary unitage of 100 IU anti-Vi IgG per ampoule of 16/138, and a relative potency of 163 IU per vial of Vi-IgG_R1, 2011_ [28]. A study of human volunteers, vaccinated with the same vaccines as this study (TCV or Vi-PS) and challenged with a high dose of *S* Typhi, showed an efficacy of 52–54.6% [24]. Although, the challenge study by Jin et al. did not identify a threshold of protection for anti-Vi IgG titres, logistic regression modelling showed that high anti-Vi IgG titres were associated with a lower probability of typhoid fever infection in this model [24]. The challenge model likely underestimates field efficacy (as discussed by Jin et al.), we therefore propose that the moderate to high level of anti-Vi IgG detected in 16/138 can be associated with a lower risk of acquiring typhoid fever, enhancing the value of 16/138 as a benchmark for assays used for the evaluation of clinical trial studies of new and existing TCV formulations.

10/126, the previous candidate IS, was included as a coded test sample and the estimated potency relative to Vi-IgG_R1, 2011_ by VaccZyme ELISA showed excellent agreement with the value reported in the previous collaborative study [15]. This confirms the outcome of the current study and further supports the use of 10/126 as a working standard. Based on the commercial ELISA, ECBS assigned a relative potency of 54 IU per ampoule of 10/126, which is equivalent to 109 IU mL^−1^ [28].

In-house ELISAs 2, 3 and 7, which coat unaided Vi to the plate surface were not robust and did not perform well in this study. These assays showed high levels of variability and poor linearity of the dose response curve.

Good concordance was seen between the VaccZyme ELISA and in-house ELISAs 1, 4 and 5. The study results indicate that the NIBSC ELISA, despite a good performance is unsuitable as a an alternative for the commercial ELISA as no agreement in relative potency estimates or ranking of coded test samples was observed between the two types of ELISA. Indeed, concordance was lacking between the commercial VaccZymeˆ ELISA and both ELISAs based on biotinylated Vi: the NIBSC ELISA and the in-house ELISA of Laboratory 6. We attribute this differentiation to the choice of coating procedures for Vi: in-house ELISAs 1, 4 and 5 use a protein to enhance the binding of native Vi to the plate surface. Whereas the modification of Vi through biotinylation seems to have altered the presentation of the Vi antigen on the plate surface, resulting in a different population of anti-Vi IgG being detected. Unfortunately, the coating procedure for Vi used in the VaccZymeˆ ELISA remains unknown and therefore this observation cannot be verified.

We conclude that either in-house ELISA 1, 4 or 5 could be an alternative for the VaccZyme ELISA. The in-house ELISA of Laboratory 1 has two advantages compared with in-house ELISA 5: the procedure was successfully transferred to Laboratory 4; and a detailed analysis of data from in-house ELISA 1 and 4 showed improved parallelism and assay precision for the study samples. Therefore, a follow-up multi laboratory study with in-house ELISA 1 will be initiated to demonstrate if this format can become the consensus assay and an alternative for the commercial ELISA.

To summarise our results: 16/138 contains a freeze-dried residue of a post-vaccination serum pool with a potency of 100 IU anti-Vi IgG per ampoule. The fill met WHO specifications, is stable and 16/138 is suitable as an IS for anti-Vi IgG (human) in various ELISA formats.

## Appendix A Supplementary data

Data Profile Data Profile

Validation_of_16-138_final_revised_amended_version.docx [41-49] Validation_of_16-138_final_revised_amended_version.docx [41-49]
