# Supplementary material for: Establishment of the first International Standard for human anti-typhoid capsular Vi polysaccharide IgG
Source: Biologicals. 2018 Nov;56:29–38. doi: 10.1016/j.biologicals.2018.09.001 (PMC6238147; doi:10.1016/j.biologicals.2018.09.001)
Supplement: Validation_of_16-138_final_revised_amended_version.docx [41-49] [file mmc2.docx]

*Supplementary tables*

Table A1: Individual assay estimates for potencies relative to 16/138

Table A2: Individual assay estimates for potencies relative to Vi-IgG _R1, 2011_

Table A1: Individual assay estimates for potencies relative to candidate IS 16/138

| ELISA  method | Laboratory  code | Sample  code | Plate ID | | | | | | GM | GCV | Number of runs |
| --- | --- | --- | --- | --- | --- | --- | --- | --- | --- | --- | --- |
|  |  |  | 1 | 2 | 3 | 4 | 5 | 6 |  |  |  |
| NIBSC | 1 | Vi-IgG_R1, 2011_ | 3.03 | 2.46 | 2.48 | 2.74 | 3.19 | 2.99 | 2.80 | 12% | 6 |
| NIBSC | 1 | A | 1.40 | 1.29 | 1.23 | 1.30 | 1.33 | 1.51 | 1.34 | 7% | 6 |
| NIBSC | 1 | B | 0.74 | 0.70 | 0.80 | 0.63 | 0.72 | 0.80 | 0.73 | 9% | 6 |
| NIBSC | 1 | C | 0.86 | 0.77 | 0.77 | 0.94 | 0.86 | 0.89 | 0.85 | 8% | 6 |
| NIBSC | 1 | D | 0.49 | 0.41 | 0.47 | 0.41 | 0.53 | 0.48 | 0.46 | 11% | 6 |
| NIBSC | 1 | E | 0.34 | 0.27 | 0.34 | 0.31 | 0.35 | 0.34 | 0.33 | 10% | 6 |
| NIBSC | 2 | Vi-IgG_R1, 2011_ | 1.56 | NL | 2.00 | 0.87 | 1.08 | 1.79 | 1.40 | 42% | 5 |
| NIBSC | 2 | A | NL | NL | 1.21 | 0.74 | 0.72 | 1.46 | 0.98 | 43% | 4 |
| NIBSC | 2 | B | 1.08 | 0.16 | 1.13 | 0.59 | NL | 0.92 | 0.64 | 123% | 5 |
| NIBSC | 2 | C | 0.74 | 2.82 | 0.90 | 0.51 | 0.50 | 1.33 | 0.93 | 93% | 6 |
| NIBSC | 2 | D | 0.15 | 0.82 | 0.16 | 0.14 | 0.13 | 0.15 | 0.19 | 104% | 6 |
| NIBSC | 2 | E | 0.31 | 0.55 | 0.37 | 0.16 | 0.15 | 0.31 | 0.46 | 388% | 6 |
| NIBSC | 3 | Vi-IgG_R1, 2011_ | *3.64* | *3.76* | *3.79* | *3.70* | *3.68* | *3.71* | *3.71* | *1%* | *6* |
| NIBSC | 3 | A | *3.68* | *3.75* | *3.80* | *3.64* | *3.66* | *3.78* | *3.72* | *2%* | *6* |
| NIBSC | 3 | B | *3.53* | *3.63* | *3.69* | *3.56* | *3.62* | *3.68* | *3.62* | *2%* | *6* |
| NIBSC | 3 | C | *3.90* | *4.11* | *4.13* | *3.95* | *4.01* | *4.13* | *4.04* | *2%* | *6* |
| NIBSC | 3 | D | *3.17* | *3.26* | *3.24* | *3.18* | *3.17* | *3.25* | *3.21* | *1%* | *6* |
| NIBSC | 3 | E | *4.12* | *4.22* | *4.26* | *4.16* | *4.20* | *4.24* | *4.20* | *1%* | *6* |
| NIBSC | 4 | Vi-IgG_R1, 2011_ | 4.99 | 3.11 | 3.12 | 3.17 | 3.97 | 3.02 | 3.50 | 22% | 6 |
| NIBSC | 4 | A | NP | 1.43 | 1.34 | 1.46 | 1.52 | 1.61 | 1.47 | 7% | 5 |
| NIBSC | 4 | B | 0.97 | 0.70 | 0.72 | 0.81 | 0.79 | 0.66 | 0.77 | 15% | 6 |
| NIBSC | 4 | C | 0.89 | 0.93 | 0.88 | 0.96 | 0.95 | 0.91 | 0.92 | 3% | 6 |
| NIBSC | 4 | D | NL | 0.43 | 0.51 | 0.57 | 0.50 | NP | 0.50 | 13% | 4 |
| NIBSC | 4 | E | 0.37 | 0.43 | 0.37 | 0.41 | 0.40 | 0.36 | 0.39 | 7% | 6 |
| NIBSC | 5 | Vi-IgG_R1, 2011_ | 3.04 | 2.49 | 2.46 | 2.70 | 2.11 | 2.99 | 2.61 | 15% | 6 |
| NIBSC | 5 | A | 1.80 | 1.46 | 1.62 | NP | NP | NP | 1.62 | 11% | 3 |
| NIBSC | 5 | B | 0.81 | 0.60 | 0.84 | 0.70 | 0.85 | 0.69 | 0.74 | 15% | 6 |
| NIBSC | 5 | C | 1.03 | 0.74 | 1.00 | 0.86 | 1.00 | 0.83 | 0.90 | 14% | 6 |
| NIBSC | 5 | D | 0.67 | 0.62 | 0.83 | 0.56 | NP | 0.67 | 0.66 | 16% | 5 |
| NIBSC | 5 | E | 0.35 | 0.32 | 0.45 | 0.38 | 0.40 | 0.39 | 0.38 | 12% | 6 |

| ELISA  method | Laboratory  code | Sample  code | Plate ID | | | | | | GM | GCV | Number of runs |
| --- | --- | --- | --- | --- | --- | --- | --- | --- | --- | --- | --- |
|  |  |  | 1 | 2 | 3 | 4 | 5 | 6 |  |  |  |
| NIBSC | 6 | Vi-IgG_R1, 2011_ | 5.09 | 2.77 | 2.92 | 2.85 | 4.60 | 5.17 | 3.75 | 36% | 6 |
| NIBSC | 6 | A | 1.78 | 1.60 | 1.61 | 1.60 | 1.79 | 1.82 | 1.70 | 6% | 6 |
| NIBSC | 6 | B | 1.04 | 1.00 | 0.94 | 1.04 | 1.11 | 1.03 | 1.03 | 6% | 6 |
| NIBSC | 6 | C | 1.23 | 1.10 | 1.15 | 1.16 | 1.31 | 1.34 | 1.21 | 8% | 6 |
| NIBSC | 6 | D | 0.27 | 0.25 | 0.25 | 0.24 | NP | 0.26 | 0.25 | 5% | 5 |
| NIBSC | 6 | E | 0.43 | 0.45 | 0.38 | 0.42 | 0.47 | 0.46 | 0.43 | 8% | 6 |
| NIBSC | 7 | Vi-IgG_R1, 2011_ | 2.33 | 1.63 | 1.49 | NP | 1.84 | NP | 1.80 | 22% | 4 |
| NIBSC | 7 | A | 1.13 | 0.88 | NP | 1.24 | 1.26 | 1.31 | 1.15 | 17% | 5 |
| NIBSC | 7 | B | 0.70 | NP | NP | NP | 0.75 | NL | 0.73 | 5% | 2 |
| NIBSC | 7 | C | 0.96 | 0.93 | 1.05 | 0.96 | 1.08 | 1.58 | 1.07 | 22% | 6 |
| NIBSC | 7 | D | 0.35 | 0.26 | 0.14 | 0.27 | NL | 0.27 | 0.25 | 42% | 5 |
| NIBSC | 7 | E | 0.49 | NP | 0.65 | 0.67 | 0.66 | 0.55 | 0.60 | 14% | 5 |
| VaccZyme | 1 | Vi-IgG_R1, 2011_ | 1.67 | 1.52 | 1.59 | 1.66 | 1.61 | 1.56 | 1.60 | 4% | 6 |
| VaccZyme | 1 | A | NL | 0.83 | 0.91 | 0.88 | 0.80 | 0.77 | 0.84 | 7% | 5 |
| VaccZyme | 1 | B | 0.29 | 0.27 | 0.28 | NP | 0.26 | 0.26 | 0.27 | 5% | 5 |
| VaccZyme | 1 | C | 0.77 | 0.69 | 0.77 | 0.70 | 0.65 | 0.63 | 0.70 | 9% | 6 |
| VaccZyme | 1 | D | 0.21 | 0.20 | 0.22 | 0.19 | 0.20 | 0.22 | 0.21 | 5% | 6 |
| VaccZyme | 1 | E | 0.46 | 0.44 | 0.47 | 0.44 | 0.41 | 0.42 | 0.44 | 6% | 6 |
| VaccZyme | 2 | Vi-IgG_R1, 2011_ | 1.93 | 2.04 | 2.12 | 1.99 | - | - | 2.02 | 4% | 4 |
| VaccZyme | 2 | A | 1.15 | 0.95 | 1.02 | 1.03 | - | - | 1.04 | 8% | 4 |
| VaccZyme | 2 | B | 0.59 | 0.44 | 0.44 | 0.44 | - | - | 0.48 | 15% | 4 |
| VaccZyme | 2 | C | 1.20 | 0.89 | 0.91 | 0.91 | - | - | 0.97 | 15% | 4 |
| VaccZyme | 2 | D | 0.25 | NP | 0.16 | 0.15 | - | - | 0.18 | 32% | 3 |
| VaccZyme | 2 | E | 0.87 | 0.53 | 0.52 | 0.53 | - | - | 0.60 | 28% | 4 |
| VaccZyme | 3 | Vi-IgG_R1, 2011_ | 1.56 | 1.52 | 1.81 | 1.50 | 1.82 | 1.42 | 1.60 | 11% | 6 |
| VaccZyme | 3 | A | 1.10 | 1.21 | 1.20 | 1.23 | 1.32 | 1.04 | 1.18 | 9% | 6 |
| VaccZyme | 3 | B | 0.44 | 0.44 | 0.45 | 0.53 | 0.50 | 0.44 | 0.47 | 8% | 6 |
| VaccZyme | 3 | C | 0.91 | 0.93 | 1.03 | 1.07 | 1.09 | 1.04 | 1.01 | 8% | 6 |
| VaccZyme | 3 | D | 0.23 | 0.25 | 0.25 | 0.26 | 0.29 | 0.22 | 0.25 | 9% | 6 |
| VaccZyme | 3 | E | 0.57 | 0.69 | 0.64 | 0.68 | 0.74 | 0.61 | 0.65 | 10% | 6 |

| ELISA  method | Laboratory  code | Sample  code | Plate ID | | | | | | GM | GCV | Number of runs |
| --- | --- | --- | --- | --- | --- | --- | --- | --- | --- | --- | --- |
|  |  |  | 1 | 2 | 3 | 4 | 5 | 6 |  |  |  |
| VaccZyme | 4 | Vi-IgG_R1, 2011_ | 1.81 | 1.32 | 2.04 | 1.45 | 1.75 | 1.32 | 1.59 | 20% | 6 |
| VaccZyme | 4 | A | 1.22 | 1.01 | 1.17 | 1.30 | 1.11 | 0.82 | 1.09 | 18% | 6 |
| VaccZyme | 4 | B | 0.40 | 0.39 | 0.47 | 0.43 | 0.44 | 0.35 | 0.41 | 11% | 6 |
| VaccZyme | 4 | C | 1.10 | 0.84 | 1.32 | 1.13 | 1.05 | 1.08 | 1.08 | 16% | 6 |
| VaccZyme | 4 | D | 0.27 | 0.28 | 0.40 | 0.37 | 0.32 | 0.24 | 0.31 | 22% | 6 |
| VaccZyme | 4 | E | 0.60 | 0.76 | 0.84 | 0.68 | 0.69 | NP | 0.71 | 14% | 5 |
| VaccZyme | 5 | Vi-IgG_R1, 2011_ | 1.25 | 1.28 | 1.86 | 1.64 | 1.14 | 1.31 | 1.39 | 20% | 6 |
| VaccZyme | 5 | A | 1.07 | 1.21 | 1.25 | 1.34 | 1.24 | 1.31 | 1.23 | 8% | 6 |
| VaccZyme | 5 | B | 0.29 | 0.33 | 0.34 | 0.34 | 0.27 | 0.31 | 0.31 | 9% | 6 |
| VaccZyme | 5 | C | 0.83 | 0.94 | 1.06 | 0.92 | 0.97 | 0.94 | 0.94 | 9% | 6 |
| VaccZyme | 5 | D | 0.30 | 0.28 | 0.36 | 0.32 | 0.29 | 0.33 | 0.31 | 9% | 6 |
| VaccZyme | 5 | E | 0.58 | 0.63 | 0.74 | 0.68 | 0.74 | 0.61 | 0.66 | 11% | 6 |
| VaccZyme | 6 | Vi-IgG_R1, 2011_ | 1.75 | 1.53 | 1.61 | 1.62 | 1.64 | - | 1.63 | 5% | 5 |
| VaccZyme | 6 | A | 1.19 | 1.20 | 1.11 | 1.33 | 1.23 | - | 1.21 | 7% | 5 |
| VaccZyme | 6 | B | NP | 0.38 | 0.37 | 0.42 | 0.40 | - | 0.39 | 6% | 4 |
| VaccZyme | 6 | C | 1.26 | 1.22 | 1.14 | 1.23 | 1.26 | - | 1.22 | 4% | 5 |
| VaccZyme | 6 | D | 0.29 | 0.28 | 0.29 | 0.30 | 0.30 | - | 0.29 | 3% | 5 |
| VaccZyme | 6 | E | 0.72 | 0.66 | 0.66 | 0.74 | 0.70 | - | 0.69 | 5% | 5 |
| VaccZyme | 7 | Vi-IgG_R1, 2011_ | 1.50 | 1.77 | 1.69 | 1.56 | 1.39 | 1.76 | 1.60 | 10% | 6 |
| VaccZyme | 7 | A | 0.88 | 1.02 | 1.03 | 0.82 | 0.62 | 1.64 | 0.96 | 38% | 6 |
| VaccZyme | 7 | B | 0.16 | 0.33 | 0.67 | 0.43 | NP | NP | 0.35 | 83% | 4 |
| VaccZyme | 7 | C | 0.90 | 1.07 | 0.97 | 0.93 | 1.06 | 1.43 | 1.05 | 18% | 6 |
| VaccZyme | 7 | D | 0.06 | 0.17 | 0.43 | 0.34 | 0.26 | NP | 0.21 | 112% | 5 |
| VaccZyme | 7 | E | 0.06 | 0.18 | 0.83 | 0.91 | 0.27 | NP | 0.30 | 206% | 5 |
| In-house | 1 | Vi-IgG_R1, 2011_ | 1.78 | 1.89 | 1.91 | 1.90 | 1.88 | 1.93 | 1.88 | 3% | 6 |
| In-house | 1 | A | 0.88 | 0.88 | 0.89 | 1.01 | 0.92 | 0.99 | 0.93 | 6% | 6 |
| In-house | 1 | B | 0.47 | 0.44 | 0.45 | 0.47 | 0.45 | 0.45 | 0.46 | 3% | 6 |
| In-house | 1 | C | 1.03 | 1.08 | 0.99 | 1.11 | 1.01 | 1.05 | 1.04 | 4% | 6 |
| In-house | 1 | D | 0.19 | 0.20 | 0.22 | 0.24 | 0.23 | 0.24 | 0.22 | 9% | 6 |
| In-house | 1 | E | 0.63 | 0.66 | 0.68 | 0.73 | 0.68 | 0.71 | 0.68 | 6% | 6 |

| ELISA  method | Laboratory  code | Sample  code | Plate ID | | | | | | GM | GCV | Number of runs |
| --- | --- | --- | --- | --- | --- | --- | --- | --- | --- | --- | --- |
|  |  |  | 1 | 2 | 3 | 4 | 5 | 6 |  |  |  |
| In-house | 2 | Vi-IgG_R1, 2011_ | 0.26 | 0.27 | - | - | - | - | 0.26 | nd | 2 |
| In-house | 2 | A | 0.62 | 0.45 | - | - | - | - | 0.53 | nd | 2 |
| In-house | 2 | B | 0.42 | 0.06 | - | - | - | - | 0.16 | nd | 2 |
| In-house | 2 | C | 1.23 | 1.01 | - | - | - | - | 1.11 | nd | 2 |
| In-house | 2 | D | 2.21 | 2.53 | - | - | - | - | 2.36 | nd | 2 |
| In-house | 2 | E | 1.68 | 1.00 | - | - | - | - | 1.30 | nd | 2 |
| In-house | 3 | Vi-IgG_R1, 2011_ | *2.35* | *2.23* | *2.17* | *2.07* | *2.15* | *2.10* | *2.18* | *5%* | *6* |
| In-house | 3 | A | *3.17* | *2.94* | *2.78* | *2.71* | *2.78* | *2.81* | *2.86* | *6%* | *6* |
| In-house | 3 | B | *2.63* | *2.51* | *2.37* | *2.22* | *2.25* | *2.26* | *2.37* | *7%* | *6* |
| In-house | 3 | C | *3.04* | *3.00* | *2.80* | *2.74* | *2.81* | *2.79* | *2.86* | *4%* | *6* |
| In-house | 3 | D | *2.04* | *1.94* | *1.85* | *1.73* | *1.73* | *1.80* | *1.85* | *7%* | *6* |
| In-house | 3 | E | *1.80* | *1.72* | *1.69* | *1.71* | *1.67* | *1.64* | *1.70* | *3%* | *6* |
| In-house | 4 | Vi-IgG_R1, 2011_ | 2.11 | 2.12 | 1.72 | 1.81 | 1.94 | 1.70 | 1.89 | 10% | 6 |
| In-house | 4 | A | 1.21 | 1.24 | 1.12 | 1.07 | 1.11 | 1.26 | 1.17 | 7% | 6 |
| In-house | 4 | B | 0.39 | 0.42 | 0.38 | 0.40 | 0.46 | 0.41 | 0.41 | 7% | 6 |
| In-house | 4 | C | 0.95 | 0.97 | 1.07 | 1.07 | 0.96 | 1.06 | 1.01 | 6% | 6 |
| In-house | 4 | D | 0.32 | 0.36 | 0.35 | 0.33 | 0.32 | 0.31 | 0.33 | 6% | 6 |
| In-house | 4 | E | 0.71 | 0.72 | 0.69 | 0.61 | 0.66 | 0.60 | 0.66 | 8% | 6 |
| In-house | 5 | Vi-IgG_R1, 2011_ | 1.60 | 1.91 | 2.16 | 1.54 | 1.47 | 1.62 | 1.70 | 16% | 6 |
| In-house | 5 | A | 1.01 | 1.12 | NP | 1.29 | 0.96 | NP | 1.09 | 14% | 4 |
| In-house | 5 | B | 0.57 | 0.49 | NP | 0.49 | 0.59 | NP | 0.53 | 10% | 4 |
| In-house | 5 | C | 0.90 | NP | 0.68 | 1.06 | 0.83 | 1.11 | 0.90 | 22% | 5 |
| In-house | 5 | D | 0.27 | NP | 0.16 | NP | 0.26 | NP | 0.23 | 34% | 3 |
| In-house | 5 | E | 0.55 | 0.46 | 0.38 | 0.50 | 0.50 | NP | 0.47 | 15% | 5 |
| In-house | 6 | Vi-IgG_R1, 2011_ | 4.82 | 5.29 | 5.32 | 4.94 | 5.42 | 5.29 | 5.18 | 5% | 6 |
| In-house | 6 | A | 1.43 | 1.50 | 1.50 | 1.49 | 1.60 | 1.52 | 1.51 | 4% | 6 |
| In-house | 6 | B | 1.26 | 1.20 | 1.17 | 1.26 | 1.34 | 1.27 | 1.25 | 5% | 6 |
| In-house | 6 | C | 1.06 | 1.07 | 1.19 | 1.13 | 1.22 | 1.22 | 1.15 | 7% | 6 |
| In-house | 6 | D | 0.11 | 0.14 | 0.14 | 0.14 | 0.14 | 0.15 | 0.14 | 9% | 6 |
| In-house | 6 | E | 0.41 | 0.42 | NP | 0.41 | 0.46 | 0.42 | 0.42 | 5% | 5 |
| In-house | 7 | Vi-IgG_R1, 2011_ | 0.79 | 1.47 | NL | NP | NP | NL | 1.08 | 55% | 2 |
| In-house | 7 | A | 0.68 | 1.19 | NL | 1.63 | NP | NP | 1.10 | 55% | 3 |
| In-house | 7 | B | 0.20 | NP | NL | NP | NP | NL | 0.20 | nd | 1 |
| In-house | 7 | C | 0.60 | NP | NL | NP | 0.90 | 0.97 | 0.80 | 29% | 3 |
| In-house | 7 | D | NP | NP | NL | 0.43 | NP | 0.41 | 0.42 | 3% | 2 |
| In-house | 7 | E | NP | 0.14 | NL | NP | NP | 0.22 | 0.18 | 37% | 2 |

GM: Geometric Mean

GCV: Geometric Coefficient of Variation

nd: not done

NP: Non-parallelism

NL: Non-linearity

Italicized entries represent original data from the participant not calculated by NIBSC

-: indicate sample not tested

Table A2: Individual assay estimates for potencies relative to U.S. reference reagent Vi-IgG_R1, 2011_

| ELISA  method | Laboratory  code | Sample  code | Plate | | | | | | GM | GCV | Number of runs |
| --- | --- | --- | --- | --- | --- | --- | --- | --- | --- | --- | --- |
|  |  |  | 1 | 2 | 3 | 4 | 5 | 6 |  |  |  |
| NIBSC | 1 | 16/138 | 0.33 | 0.41 | 0.40 | 0.36 | 0.31 | 0.33 | 0.36 | 12% | 6 |
| NIBSC | 1 | A | 0.48 | 0.45 | 0.46 | 0.49 | 0.43 | NP | 0.46 | 5% | 5 |
| NIBSC | 1 | B | 0.25 | 0.26 | 0.30 | 0.23 | 0.22 | 0.25 | 0.25 | 11% | 6 |
| NIBSC | 1 | C | 0.29 | 0.28 | 0.29 | 0.35 | 0.27 | 0.28 | 0.29 | 10% | 6 |
| NIBSC | 1 | D | 0.16 | 0.16 | 0.19 | 0.15 | NP | 0.15 | 0.16 | 9% | 5 |
| NIBSC | 1 | E | 0.11 | NP | 0.13 | 0.11 | 0.11 | 0.11 | 0.12 | 8% | 5 |
| NIBSC | 2 | 16/138 | 0.64 | NL | 0.50 | 1.15 | 0.92 | 0.56 | 0.72 | 42% | 5 |
| NIBSC | 2 | A | NL | NL | 0.65 | 0.85 | 0.65 | 0.78 | 0.73 | 15% | 4 |
| NIBSC | 2 | B | 0.72 | NL | 0.60 | 0.68 | NL | 0.50 | 0.62 | 17% | 4 |
| NIBSC | 2 | C | 0.49 | NL | 0.48 | 0.59 | 0.45 | NP | 0.50 | 12% | 4 |
| NIBSC | 2 | D | 0.09 | NL | NP | 0.15 | 0.12 | 0.09 | 0.11 | 30% | 4 |
| NIBSC | 2 | E | 0.20 | NL | 0.20 | 0.18 | 0.14 | 0.18 | 0.18 | 15% | 5 |
| NIBSC | 3 | 16/138 | *0.27* | *0.27* | *0.26* | *0.27* | *0.27* | *0.27* | *0.27* | *1%* | *6* |
| NIBSC | 3 | A | *1.01* | *1.00* | *1.00* | *0.98* | *0.99* | *1.02* | *1.00* | *1%* | *6* |
| NIBSC | 3 | B | *0.97* | *0.96* | *0.98* | *0.96* | *0.98* | *0.99* | *0.97* | *1%* | *6* |
| NIBSC | 3 | C | *1.07* | *1.09* | *1.09* | *1.07* | *1.09* | *1.11* | *1.09* | *2%* | *6* |
| NIBSC | 3 | D | *0.87* | *0.87* | *0.86* | *0.86* | *0.86* | *0.88* | *0.86* | *1%* | *6* |
| NIBSC | 3 | E | *1.13* | *1.12* | *1.13* | *1.12* | *1.14* | *1.14* | *1.13* | *1%* | *6* |
| NIBSC | 4 | 16/138 | 0.20 | 0.32 | 0.32 | 0.32 | 0.25 | 0.33 | 0.29 | 22% | 6 |
| NIBSC | 4 | A | NP | 0.47 | 0.44 | 0.46 | 0.40 | 0.57 | 0.47 | 14% | 5 |
| NIBSC | 4 | B | 0.19 | 0.23 | 0.23 | 0.26 | 0.20 | 0.22 | 0.22 | 11% | 6 |
| NIBSC | 4 | C | 0.18 | 0.31 | 0.29 | 0.30 | 0.24 | 0.31 | 0.27 | 24% | 6 |
| NIBSC | 4 | D | NL | 0.14 | 0.16 | 0.18 | 0.12 | 0.16 | 0.15 | 17% | 5 |
| NIBSC | 4 | E | 0.08 | 0.14 | 0.12 | 0.13 | 0.10 | 0.12 | 0.11 | 25% | 6 |
| NIBSC | 5 | 16/138 | 0.33 | 0.40 | 0.41 | 0.37 | 0.47 | 0.33 | 0.38 | 15% | 6 |
| NIBSC | 5 | A | 0.66 | 0.63 | 0.65 | NP | NP | NP | 0.65 | 2% | 3 |
| NIBSC | 5 | B | 0.27 | 0.22 | 0.34 | 0.26 | 0.40 | 0.23 | 0.28 | 26% | 6 |
| NIBSC | 5 | C | 0.35 | NP | 0.40 | 0.31 | 0.47 | 0.29 | 0.36 | 22% | 5 |
| NIBSC | 5 | D | NP | 0.22 | 0.33 | 0.21 | NP | 0.23 | 0.24 | 24% | 4 |
| NIBSC | 5 | E | 0.11 | 0.12 | 0.18 | 0.14 | 0.19 | 0.13 | 0.14 | 23% | 6 |
| NIBSC | 6 | 16/138 | 0.20 | 0.36 | 0.34 | 0.35 | 0.22 | 0.19 | 0.27 | 36% | 6 |
| NIBSC | 6 | A | 0.35 | 0.57 | 0.55 | 0.56 | 0.39 | 0.35 | 0.45 | 27% | 6 |
| NIBSC | 6 | B | 0.21 | 0.36 | 0.32 | 0.36 | 0.24 | 0.20 | 0.27 | 31% | 6 |
| NIBSC | 6 | C | 0.24 | 0.40 | 0.39 | 0.41 | 0.28 | 0.26 | 0.32 | 27% | 6 |
| NIBSC | 6 | D | 0.05 | 0.09 | 0.09 | 0.08 | 0.06 | 0.05 | 0.07 | 30% | 6 |
| NIBSC | 6 | E | 0.08 | 0.17 | 0.13 | 0.15 | 0.10 | 0.09 | 0.12 | 32% | 6 |

| ELISA  method | Laboratory  code | Sample  code | Plate ID | | | | | | GM | GCV | Number of runs |
| --- | --- | --- | --- | --- | --- | --- | --- | --- | --- | --- | --- |
|  |  |  | 1 | 2 | 3 | 4 | 5 | 6 |  |  |  |
| NIBSC | 7 | 16/138 | 0.43 | 0.61 | 0.67 | NP | 0.54 | NP | 0.56 | 22% | 4 |
| NIBSC | 7 | A | 0.48 | 0.57 | 0.61 | NL | 0.59 | NL | 0.56 | 11% | 4 |
| NIBSC | 7 | B | 0.31 | 0.40 | NP | NP | 0.45 | NL | 0.38 | 22% | 3 |
| NIBSC | 7 | C | 0.43 | 0.60 | 0.73 | NP | 0.64 | NP | 0.59 | 25% | 4 |
| NIBSC | 7 | D | 0.14 | 0.20 | 0.13 | 0.22 | NL | NL | 0.17 | 28% | 4 |
| NIBSC | 7 | E | 0.21 | NP | 0.49 | NP | 0.40 | NL | 0.34 | 56% | 3 |
| VaccZyme | 1 | 16/138 | 0.60 | 0.66 | 0.63 | 0.60 | 0.62 | 0.64 | 0.62 | 4% | 6 |
| VaccZyme | 1 | A | NL | 0.58 | 0.59 | 0.55 | 0.50 | 0.54 | 0.55 | 7% | 5 |
| VaccZyme | 1 | B | 0.17 | 0.18 | 0.17 | 0.15 | 0.16 | 0.17 | 0.17 | 7% | 6 |
| VaccZyme | 1 | C | 0.47 | 0.48 | 0.49 | 0.43 | 0.40 | 0.44 | 0.45 | 8% | 6 |
| VaccZyme | 1 | D | 0.12 | 0.13 | 0.13 | 0.11 | 0.12 | 0.14 | 0.13 | 9% | 6 |
| VaccZyme | 1 | E | 0.27 | 0.30 | 0.29 | 0.26 | 0.24 | 0.28 | 0.27 | 8% | 6 |
| VaccZyme | 2 | 16/138 | 0.52 | 0.49 | 0.47 | 0.50 | - | - | 0.50 | 4% | 4 |
| VaccZyme | 2 | A | 0.59 | 0.46 | 0.49 | 0.51 | - | - | 0.51 | 10% | 4 |
| VaccZyme | 2 | B | 0.30 | 0.21 | 0.21 | 0.22 | - | - | 0.23 | 19% | 4 |
| VaccZyme | 2 | C | 0.61 | 0.43 | 0.44 | 0.45 | - | - | 0.48 | 18% | 4 |
| VaccZyme | 2 | D | 0.13 | NP | 0.07 | 0.07 | - | - | 0.09 | 39% | 3 |
| VaccZyme | 2 | E | 0.43 | 0.25 | 0.25 | 0.26 | - | - | 0.29 | 31% | 4 |
| VaccZyme | 3 | 16/138 | 0.64 | 0.66 | 0.55 | 0.67 | 0.55 | 0.71 | 0.63 | 11% | 6 |
| VaccZyme | 3 | A | 0.70 | 0.81 | 0.65 | 0.82 | 0.72 | 0.70 | 0.73 | 9% | 6 |
| VaccZyme | 3 | B | 0.28 | 0.29 | 0.24 | 0.37 | 0.28 | 0.30 | 0.29 | 15% | 6 |
| VaccZyme | 3 | C | 0.58 | 0.62 | 0.56 | 0.72 | 0.60 | 0.71 | 0.63 | 11% | 6 |
| VaccZyme | 3 | D | 0.15 | 0.17 | 0.14 | 0.19 | 0.16 | 0.16 | 0.16 | 12% | 6 |
| VaccZyme | 3 | E | 0.36 | 0.46 | 0.34 | 0.44 | 0.41 | 0.42 | 0.40 | 12% | 6 |
| VaccZyme | 4 | 16/138 | 0.55 | 0.76 | 0.49 | 0.69 | 0.57 | 0.76 | 0.63 | 20% | 6 |
| VaccZyme | 4 | A | 0.66 | 0.85 | 0.60 | 0.89 | 0.65 | 0.60 | 0.70 | 19% | 6 |
| VaccZyme | 4 | B | 0.22 | 0.30 | 0.23 | 0.29 | 0.25 | 0.27 | 0.26 | 13% | 6 |
| VaccZyme | 4 | C | 0.60 | 0.70 | 0.67 | 0.78 | 0.62 | 0.76 | 0.68 | 11% | 6 |
| VaccZyme | 4 | D | 0.15 | 0.22 | 0.20 | 0.25 | 0.18 | 0.19 | 0.19 | 19% | 6 |
| VaccZyme | 4 | E | 0.33 | 0.63 | 0.42 | 0.46 | 0.39 | 0.42 | 0.43 | 24% | 6 |
| VaccZyme | 5 | 16/138 | 0.80 | 0.78 | 0.54 | 0.61 | 0.88 | 0.76 | 0.72 | 20% | 6 |
| VaccZyme | 5 | A | 0.82 | 0.95 | 0.65 | 0.78 | 0.96 | 0.94 | 0.84 | 17% | 6 |
| VaccZyme | 5 | B | 0.23 | 0.27 | 0.18 | 0.20 | 0.25 | 0.24 | 0.23 | 15% | 6 |
| VaccZyme | 5 | C | 0.64 | 0.75 | 0.55 | 0.54 | 0.77 | 0.70 | 0.65 | 16% | 6 |
| VaccZyme | 5 | D | 0.24 | 0.23 | 0.19 | 0.20 | 0.26 | 0.26 | 0.23 | 14% | 6 |
| VaccZyme | 5 | E | 0.45 | 0.49 | 0.39 | 0.40 | 0.60 | 0.47 | 0.46 | 17% | 6 |
| VaccZyme | 6 | 16/138 | 0.57 | 0.65 | 0.62 | 0.62 | 0.61 | - | 0.61 | 5% | 5 |
| VaccZyme | 6 | A | 0.68 | 0.80 | 0.69 | 0.83 | 0.75 | - | 0.75 | 9% | 5 |
| VaccZyme | 6 | B | NP | 0.25 | 0.23 | 0.25 | 0.24 | - | 0.24 | 4% | 4 |
| VaccZyme | 6 | C | 0.73 | 0.81 | 0.71 | 0.76 | 0.77 | - | 0.76 | 5% | 5 |
| VaccZyme | 6 | D | NP | 0.18 | 0.18 | 0.18 | 0.18 | - | 0.18 | 1% | 4 |
| VaccZyme | 6 | E | NP | 0.44 | 0.41 | 0.45 | 0.43 | - | 0.43 | 4% | 4 |

| ELISA  method | Laboratory  code | Sample  code | Plate ID | | | | | | GM | GCV | Number of runs |
| --- | --- | --- | --- | --- | --- | --- | --- | --- | --- | --- | --- |
|  |  |  | 1 | 2 | 3 | 4 | 5 | 6 |  |  |  |
| VaccZyme | 7 | 16/138 | 0.67 | 0.56 | 0.59 | 0.64 | 0.72 | 0.57 | 0.62 | 10% | 6 |
| VaccZyme | 7 | A | 0.57 | 0.60 | 0.66 | 0.55 | 0.46 | 0.81 | 0.60 | 21% | 6 |
| VaccZyme | 7 | B | 0.11 | 0.18 | 0.41 | 0.27 | 0.27 | NP | 0.23 | 65% | 5 |
| VaccZyme | 7 | C | 0.58 | 0.63 | 0.61 | 0.62 | 0.77 | 0.72 | 0.65 | 11% | 6 |
| VaccZyme | 7 | D | 0.04 | 0.10 | 0.25 | 0.22 | 0.20 | NP | 0.13 | 111% | 5 |
| VaccZyme | 7 | E | 0.04 | 0.10 | 0.51 | 0.61 | 0.20 | NP | 0.19 | 210% | 5 |
| In-house | 1 | 16/138 | 0.56 | 0.53 | 0.52 | 0.53 | 0.53 | 0.52 | 0.53 | 3% | 6 |
| In-house | 1 | A | 0.47 | 0.45 | 0.45 | 0.53 | 0.48 | 0.49 | 0.48 | 6% | 6 |
| In-house | 1 | B | 0.26 | 0.23 | 0.23 | 0.24 | 0.24 | 0.23 | 0.24 | 6% | 6 |
| In-house | 1 | C | 0.55 | 0.55 | 0.51 | 0.58 | 0.52 | 0.52 | 0.54 | 5% | 6 |
| In-house | 1 | D | 0.11 | 0.11 | 0.12 | 0.13 | 0.12 | 0.12 | 0.12 | 6% | 6 |
| In-house | 1 | E | 0.34 | 0.33 | 0.35 | 0.38 | 0.35 | 0.35 | 0.35 | 5% | 6 |
| In-house | 2 | 16/138 | 3.84 | 3.77 | - | - | - | - | 3.80 | nd | 2 |
| In-house | 2 | A | 2.25 | 1.63 | - | - | - | - | 1.92 | nd | 2 |
| In-house | 2 | B | 1.58 | NP | - | - | - | - | 1.58 | nd | 2 |
| In-house | 2 | C | 4.40 | 3.46 | - | - | - | - | 3.90 | nd | 2 |
| In-house | 2 | D | 8.58 | 8.55 | - | - | - | - | 8.56 | nd | 2 |
| In-house | 2 | E | 5.96 | 3.41 | - | - | - | - | 4.51 | nd | 2 |
| In-house | 3 | 16/138 | *0.43* | *0.45* | *0.46* | *0.48* | *0.46* | *0.48* | *0.46* | *5%* | *6* |
| In-house | 3 | A | *1.35* | *1.32* | *1.28* | *1.31* | *1.29* | *1.34* | *1.31* | *2%* | *6* |
| In-house | 3 | B | *1.12* | *1.12* | *1.09* | *1.07* | *1.05* | *1.08* | *1.09* | *3%* | *6* |
| In-house | 3 | C | *1.29* | *1.34* | *1.29* | *1.32* | *1.31* | *1.33* | *1.31* | *2%* | *6* |
| In-house | 3 | D | *0.87* | *0.87* | *0.85* | *0.83* | *0.80* | *0.86* | *0.85* | *3%* | *6* |
| In-house | 3 | E | *0.76* | *0.77* | *0.78* | *0.83* | *0.78* | *0.78* | *0.78* | *3%* | *6* |
| In-house | 4 | 16/138 | 0.47 | 0.47 | 0.58 | 0.55 | 0.52 | 0.59 | 0.53 | 10% | 6 |
| In-house | 4 | A | 0.56 | 0.56 | 0.67 | 0.58 | 0.54 | NP | 0.58 | 9% | 5 |
| In-house | 4 | B | 0.19 | 0.19 | 0.22 | 0.22 | 0.23 | 0.25 | 0.22 | 12% | 6 |
| In-house | 4 | C | 0.44 | 0.45 | 0.65 | 0.58 | 0.47 | 0.62 | 0.53 | 19% | 6 |
| In-house | 4 | D | 0.15 | 0.17 | 0.21 | 0.19 | 0.16 | 0.19 | 0.18 | 12% | 6 |
| In-house | 4 | E | 0.33 | 0.33 | 0.41 | 0.34 | 0.33 | 0.36 | 0.35 | 9% | 6 |
| In-house | 5 | 16/138 | 0.62 | 0.52 | 0.46 | 0.65 | 0.68 | 0.62 | 0.59 | 16% | 6 |
| In-house | 5 | A | 0.62 | 0.54 | 0.50 | 0.84 | 0.65 | NP | 0.62 | 22% | 5 |
| In-house | 5 | B | 0.35 | 0.25 | 0.29 | 0.32 | 0.40 | 0.26 | 0.31 | 20% | 6 |
| In-house | 5 | C | 0.55 | NP | 0.31 | 0.69 | 0.56 | 0.65 | 0.53 | 37% | 5 |
| In-house | 5 | D | 0.17 | 0.11 | 0.08 | NP | 0.18 | NP | 0.13 | 48% | 4 |
| In-house | 5 | E | 0.34 | 0.23 | 0.18 | 0.32 | 0.34 | 0.25 | 0.27 | 30% | 6 |
| In-house | 6 | 16/138 | 0.21 | 0.19 | 0.19 | 0.20 | 0.18 | 0.19 | 0.19 | 5% | 6 |
| In-house | 6 | A | 0.30 | 0.29 | 0.29 | 0.30 | 0.29 | 0.29 | 0.29 | 2% | 6 |
| In-house | 6 | B | 0.26 | 0.23 | 0.22 | 0.25 | 0.25 | 0.24 | 0.24 | 7% | 6 |
| In-house | 6 | C | 0.22 | 0.20 | NP | 0.23 | 0.22 | 0.23 | 0.22 | 5% | 5 |
| In-house | 6 | D | 0.02 | 0.03 | 0.03 | 0.03 | 0.02 | 0.03 | 0.03 | 8% | 6 |
| In-house | 6 | E | 0.08 | 0.08 | NP | 0.08 | 0.08 | 0.08 | 0.08 | 3% | 5 |
| In-house | 7 | 16/138 | 1.26 | 0.68 | NL | NP | NP | NL | 0.93 | 55% | 2 |
| In-house | 7 | A | 0.84 | 0.76 | NL | NP | NP | NL | 0.80 | 8% | 2 |
| ELISA  method | Laboratory  code | Sample  code | Plate ID | | | | | | GM | GCV | Number of runs |
|  |  |  | 1 | 2 | 3 | 4 | 5 | 6 |  |  |  |
| In-house | 7 | B | 0.27 | NP | NL | 0.42 | 0.43 | NL | 0.36 | 29% | 3 |
| In-house | 7 | C | 0.75 | NP | NL | NP | 0.75 | NL | 0.75 | nd | 2 |
| In-house | 7 | D | NP | NP | NL | NP | NP | NL | nd | nd | 0 |
| In-house | 7 | E | NP | 0.11 | NL | NP | NP | NL | 0.11 | nd | 1 |

GM: Geometric Mean

GCV: Geometric Coefficient of Variation

nd: not done

NP: Non-parallelism

NL: Non-linearity

Italicized entries represent original data from the participant not calculated by NIBSC

-: indicates sample not tested
